# When the Balance Tips: Dysregulation of Mitochondrial Dynamics as a Culprit in Disease

**DOI:** 10.3390/ijms22094617

**Published:** 2021-04-28

**Authors:** Styliana Kyriakoudi, Anthi Drousiotou, Petros P. Petrou

**Affiliations:** 1 Department of Biochemical Genetics, The Cyprus Institute of Neurology and Genetics, P.O. Box 23462, Nicosia 1683, Cyprus; stylianak@cing.ac.cy (S.K.); anthidr@cing.ac.cy (A.D.); 2 Cyprus School of Molecular Medicine, P.O. Box 23462, Nicosia 1683, Cyprus

**Keywords:** mitochondrial dynamics, fusion, fission, pathology

## Abstract

Mitochondria are dynamic organelles, the morphology of which is tightly linked to their functions. The interplay between the coordinated events of fusion and fission that are collectively described as mitochondrial dynamics regulates mitochondrial morphology and adjusts mitochondrial function. Over the last few years, accruing evidence established a connection between dysregulated mitochondrial dynamics and disease development and progression. Defects in key components of the machinery mediating mitochondrial fusion and fission have been linked to a wide range of pathological conditions, such as insulin resistance and obesity, neurodegenerative diseases and cancer. Here, we provide an update on the molecular mechanisms promoting mitochondrial fusion and fission in mammals and discuss the emerging association of disturbed mitochondrial dynamics with human disease.

## 1. Introduction

Mitochondria represent a tubular, remarkably dynamic system of membrane-bound cell organelles that were first observed in high-resolution electron micrographs in the early 1950s [1]. Since their endosymbiotic biogenesis through the fateful integration of an *alpha*-proteobacterium within an archaeal host cell [2], mitochondria have become indispensable constituents of multicellular life. They are responsible for the production of the chemical energy required for cell metabolism through oxidative phosphorylation (OXPHOS) in the form of adenosine triphosphate (ATP) and thus, are often called the “energy factories” of the cell [3,4]. In the 1890s, the microbiologist Carl Benda coined the term mitochondrion from the Greek words “mitos”, which means “thread”, and “chondrion”, which means “small granule”, as he perceived, under a microscope, hundreds of tiny bodies with the tendency to form long chains in the cytoplasm of eukaryotic cells [5].

As a result of their endosymbiotic origin, mitochondria carry their own genome, which is denoted as mtDNA and encodes 13 components of the OXPHOS system that are synthesized by ribosomes present within mitochondria. The remaining subunits of the system, as well as proteins required for mtDNA replication, transcription and translation, are encoded by nuclear genes [6]. Structurally, mitochondria consist of an outer membrane that encloses the entire content of the organelle and an inner membrane that houses the OXPHOS system and delineates the mitochondrial matrix. The inner mitochondrial membrane (IMM) has a larger surface area than the outer mitochondrial membrane (OMM) and is characterized by a specific spatial arrangement consisting of a series of invaginations known as “cristae”, which extend into the matrix. Between the outer and inner membranes, there is an enclosed compartment, referred to as the intermembrane space [7].

As highly dynamic structures, mitochondria undergo morphological changes and spatial rearrangements in order to adapt to cellular demands and to maintain energy homeostasis. These changes are modulated through the coordinated cycles of mitochondrial fusion and fission, commonly referred to as mitochondrial dynamics, which control mitochondrial number, size, shape and distribution within the cell [8,9]. The delicate equilibrium between fusion and fission confers important benefits that are brought about through the exchange of mitochondrial content, maintenance of their genome and segregation of dysfunctional organelles that are targeted for autophagic degradation [10], a mitochondrial quality-control process known as mitophagy. This selective form of autophagy acts in concert with mitochondrial biogenesis for the control of mitochondrial turnover. Mitophagy is a crucial process in maintaining mitochondrial homeostasis, and aberrant sequestration of dysfunctional mitochondria due to impaired mitochondrial fission contributes to disease development [11,12].

In mammals, mitochondrial dynamics are mainly progressed by large GTPases that belong to the dynamin superfamily. In particular, mitochondrial fusion requires three key proteins, mitofusin 1 and 2 (MFN1 and MFN2) and optic atrophy 1 (OPA1) while mitochondrial fission is promoted by the dynamin-related-like protein 1 (DRP1) [13,14,15,16]. Dysregulation of these processes results in an abnormally fused or fragmented mitochondrial network that is unable to respond to cellular energy demands. An increasingly large number of mutations affecting the function of the aforementioned core proteins of the mitochondrial dynamics machinery have been associated with perturbed mitochondrial morphology [17,18,19,20,21] and a broad spectrum of human diseases [22,23].

In the present review, we discuss the dynamic nature of mitochondria and summarize the current knowledge with regards to the molecular mechanisms of mitochondrial fusion and fission in mammals. In addition, perturbations in the dynamic transitions of the mitochondrial network caused by aberrant fusion and fission will be highlighted in the context of representative human diseases.

## 2. Mitochondrial Fusion in Mammals

### 2.1. The Fusion Machinery

As previously mentioned, under physiological conditions, mitochondrial dynamics are regulated by the balanced interplay between the opposing processes of fusion and fission. The identification of several key molecules involved in mitochondrial fusion provided important insights into the molecular mechanisms underlying this process [24]. The term mitochondrial fusion signifies the merging of two originally distinct mitochondria into a single unit by a two-step process. This complex regulatory process involves the merging of the OMM and the IMM in two functionally distinct events [25]. Within a cell, mitochondria constantly migrate along the cytoskeleton to subcellular regions of high energy demand and, during migration, nearby mitochondria can fuse together, producing elongated interconnected tubules [26]. Mitochondrial connectivity that is enabled by fusion allows genetic coupling between mitochondria and confers “functional complementation” by compensating for the defective components of a damaged organelle. In addition, the energetic demands of the cell are covered through the exchange and distribution of mitochondrial material favored by the physical integration of the mitochondrial content [27].

**Figure 1:**
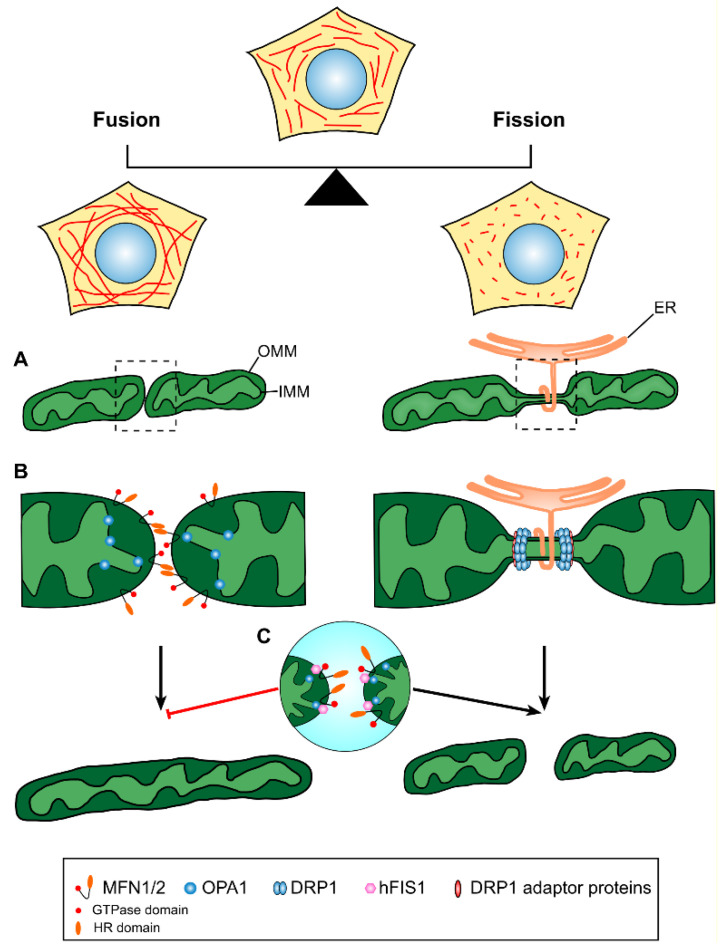
Schematic illustration of mitochondrial fusion and fission. Mitochondria are dynamic organelles undergoing coordinated cycles of mitochondrial fusion and fission. A balance in the processes of mitochondrial dynamics maintains mitochondrial network homeostasis, which is central to proper cell function. (**A**) Mitochondrial fusion (left) involves the merging of two originally distinct mitochondria into a single organelle, while mitochondrial fission (right) describes the division of a single mitochondrion in two or more individual mitochondrial units, a process that involves a membrane preconstriction step at the ER–mitochondria contact sites. (**B**) OMM tethering and fusion are promoted by the formation of MFN1/MFN2 homotypic or heterotypic complexes and the physical interaction of the mitofusins GTPase and HR domains, while IMM fusion is mediated by the inner-membrane-anchored protein OPA1. Mitochondrial fission is mainly regulated by the DRP1 protein, which is recruited to the mitochondrial division site by interacting with the adaptor proteins MFF, MiD49 and MiD51. As indicated, the ER has a prominent role in delimiting the exact site of mitochondrial division by wrapping itself around the mitochondrial tubule prior to the oligomerization of multiple DRP1 molecules in a ring-shaped structure that further constricts the membrane and reinforces mitochondrial scission. (**C**) hFIS1 interacts with the fusion mediators MFN1, MFN2 and OPA1, blocks their GTPase function and shifts the balance of mitochondrial dynamics towards fission. OMM, outer mitochondrial membrane; IMM, inner mitochondrial membrane; HR, heptad repeat.

The *Drosophila* fuzzy onions (*Fzo*) was the first mitochondrial-fusion-related gene to be identified [28]. In mammals, the key molecules implicated in the fusion process are the mitofusins (MFN1 and MFN2), which are membrane-anchored proteins promoting OMM fusion, and the OPA1 protein, which mediates the fusion of the IMM (Figure 1). Human mitofusins share approximately 80% sequence similarity and are about 50% similar to *Drosophila*’s *Fzo* [14,15]. As predicted by their amino acid sequence, MFN1 and MFN2 are structurally similar and display a multidomain configuration. The N-terminal region of the proteins contains a GTPase domain followed by a hydrophobic heptad (7-residue) repeat region, termed HR1. The C-terminal region contains a second heptad repeat domain, termed HR2, while a long transmembrane domain lies between the two HR domains [29]. Both MFN1 and MFN2 are targeted to the OMM, however, MFN2 is also localized on endoplasmic reticulum (ER) membranes, where it mediates the physical association between the ER and mitochondria [30]. Mitofusins are functionally redundant, and it has been shown that cells lacking both MFN proteins can be fully rescued by overexpressing either protein, clearly indicating their common biological role [14]. Furthermore, it has been demonstrated that the ablation of either MFN1 or MFN2 results in reduced frequency of mitochondrial fusion events in mouse embryonic fibroblasts, while the absence of both MFN1 and MFN2 leads to a complete loss of mitochondrial fusion with deleterious consequences on cellular function [31].

The *OPA1* gene encodes the mammalian homologue of the mitochondrial genome maintenance 1 (MGM1) protein that was originally described in yeast [32]. Like mitofusins, OPA1 is a critical determinant of mitochondrial fusion and is specifically responsible for the fusion of the IMM. Although OPA1 localization within mitochondria was a matter of debate for several years, with studies supporting the presence of the protein either on the OMM [32] or in the mitochondrial matrix [33], ensuing studies indicated that OPA1 is inserted into the IMM through an N-terminal matrix-targeting signal followed by a transmembrane domain, with the major part of the protein facing the intermembrane space [34,35,36]. Several studies demonstrated that the loss of OPA1 expression in mammalian cells leads to mitochondrial fragmentation and dysfunction, as well as to disorganized cristae as a result of impaired fusion, whereas OPA1 overexpression results in mitochondrial elongation [31,37,38]. Even though the crystal structure of OPA1 has not yet been resolved, a predicted three-dimensional model of the protein revealed an evolutionarily conserved C-terminal region consisting of multiple functional domains and an *alpha*-helix-rich N-terminal region that lacks specific domains. The functional domains of the C-terminus include a GTPase domain, a middle domain, a pleckstrin-homology region and a GTPase effector domain [39].

### 2.2. Molecular Mechanisms of Mitochondrial Fusion

The fusion of biological membranes is a generic cellular process that occurs during several vesicular trafficking processes. Endosome–lysosome fusion, synaptic vesicle fusion and ER to Golgi tethering are a few examples of biological membrane merging, all sharing a common set of steps promoting fusion. At first, a close apposition of membranes known as membrane “docking” is facilitated through the interaction of proteins on opposing membranes and the assembly of protein complexes. During the second step of contact, the bridging of the two membrane compartments takes place, driven by the assembly of helical bundles that brings membranes into closer proximity, thereby promoting their fusion [40]. It has been suggested that the common steps of tethering, docking and merging of biological membranes also apply to the fusion between mitochondria, with the MFN1 and MFN2 proteins playing a crucial role during the initial contact. Located on the OMM, MFN1 and MFN2 initiate the early stage of mitochondrial tethering by acting in trans on adjacent mitochondria. It has been shown that mitofusins are capable of forming homotypic oligomers, as well as heterotypic complexes, thus acting individually or synergistically to promote mitochondrial fusion. This observation further supports the idea that mitochondrial fusion requires that mitofusin complexes be located on adjacent mitochondria [14,31]. Immunoprecipitation assays and structural studies revealed that the HR2 domains at the C-terminus of either MFN1 or MFN2 can form homotypic or heterotypic complexes by folding into a dimeric antiparallel 9.5 nm long coiled coil. This large interface promotes the tethering of adjacent mitochondria by leaving a small gap between the opposing membranes. While in this trapped stage, adjacent mitochondria are driven to complete fusion by a subsequent conformational change involving the GTPase domain. Mutations that disrupt the structure of the HR2 domain of MFN2 resulted in defective mitochondrial morphology, demonstrating that this specific domain is essential for the activity of mitofusins during mitochondrial fusion [41]. Additional structural data revealed that GTP hydrolysis induces conformational changes in MFN1 that are crucial for GTPase domain dimerization. Furthermore, disruption of this domain abolishes MFN1 fusogenic function [42].

Despite the fact that the fusion of the outer and inner mitochondrial membranes are separate events that are mechanistically distinct, the fusion machineries must be coordinated to complete fusion [25]. To date, most of the mitochondrial components mediating inner-membrane fusion are still unknown, however, the localization of OPA1 in the intermembrane space close to the cristae supports the notion that it exhibits a salient function in IMM fusion and cristae morphology [10,34]. OPA1 is encoded by at least eight different spliced variants that produce two distinct isoforms of the protein: the unprocessed long isoform (L-OPA1), which is anchored to the inner membrane via the N-terminal domain, and the proteolytically cleaved short isoform (s-OPA1) which lacks the transmembrane domain and remains soluble in the intermembrane space. The proteolytic processing of OPA1 is suggested to represent a checkpoint of mitochondrial fusion and is primarily affected by the membrane potential [43,44]. Interestingly, respiratory chain failure and the loss of membrane potential result in enhanced proteolytic cleavage of L-OPA1 and accumulation of the short isoform, thus leading to the attenuation of mitochondrial fusion [43,45]. Several studies suggest that a dimer consisting of both long and short forms of OPA1 is required for efficient fusion, whereas long or short isoforms have little activity on their own [43,46,47]. In concert with this, a recent study reports that s-OPA1 is capable of mediating IMM tethering but is not sufficient for membrane docking alone, while L-OPA1 can hemifuse the bilayers but is unable to finalize the fusion [48]. All together, these observations support that a stoichiometric balance between L-OPA1 and s-OPA1 is important for efficient mitochondrial fusion. A further study proposed the existence of a minimal IMM fusion machinery composed of L-OPA1 and the mitochondrial-specific phospholipid cardiolipin. Located on opposing membranes, recombinant L-OPA1 and cardiolipin were shown to form a heterotypic complex that is sufficient to drive membrane fusion. It has been suggested that the formation of this minimal fusion complex serves as the initial step of IMM fusion that occurs independently of GTP and primes the subsequent GTP-dependent fusion step, which is completed in the presence of s-OPA1 [49]. Despite the reported findings of the above studies, the lack of purified active L-OPA1 protein raises the need for additional studies to establish a more detailed mechanistic model of inner-membrane fusion.

## 3. Mitochondrial Fission in Mammals

### 3.1. The Fission Machinery

Mitochondrial fission describes the division of one mitochondrion into separate organelles through a multistep process in response to cellular cues. Fragmentation of the mitochondrial network can occur upon stimulation of fission activity and/or inhibition of mitochondrial fusion. As in the case of mitochondrial fusion, the core components of the fission machinery were originally identified in yeast [50,51], while later studies identified the dynamin-related protein 1 (DRP1) as the main effector of mitochondrial fission in mammals [16,52,53]. This is demonstrated by the fact that mutations in *DRP1* result in hyperfused mitochondrial tubules secondary to the inhibition of mitochondrial fission [16,54]. DRP1 is an evolutionarily conserved, primarily cytosolic GTPase that is recruited to the OMM to drive membrane constriction and eventually mitochondrial fission. In its soluble form, DRP1 exists in dimers, tetramers and other oligomeric states [55]. It is composed of the typical units of dynamin-family GTPases, including an N-terminal GTPase domain followed by a central element and a C-terminal GTPase effector domain. The crystal structure of the protein further confirms the presence of a bundle-signaling element (BSE) located on top of a helical stalk [56]. 

### 3.2. Molecular Mechanisms of Mitochondrial Fission

Although the precise scission mechanism remains elusive, it has been shown that the initial step of mitochondrial fission begins with the translocation of soluble DRP1 to the OMM, where it binds to membrane-anchored receptors, oligomerizes and constricts the membrane at the fission site by forming a ring-like structure [57] (Figure 1). The recruitment of DRP1 at the division site was shown to facilitate the formation of higher-order helical structures that initiate the constriction of both inner and outer membranes and finally complete mitochondrial division upon GTP hydrolysis [58]. Once the helical structure completes a turn, the GTPase domains of adjacent rungs come in close proximity and trigger GTP hydrolysis, which in turn induces further conformational changes that drive constriction [59]. According to contradicting studies, two possible models for DRP1 recruitment at fission sites have been proposed, termed de novo assembly and targeted equilibrium. In the de novo assembly model, cytosolic DRP1 is directly recruited and oligomerized at specific fission sites immediately before division in response to fission signals [60,61]. On the other hand, targeted equilibrium implies that DRP1 dimers or oligomers are in constant balance between soluble and mitochondrially anchored pools. According to this model, DRP1 recruitment and oligomerization is progressively achieved through the incorporation of additional mitochondrially anchored DRP1 units, eventually forming a mature-sized DRP1 complex. These oligomers are motile and translocate along the mitochondrial membrane until fission signals target the complex to the exact division site. It has been shown that the mitochondrial accumulation of actin filaments precedes DRP1 assembly at fission sites. Moreover, the binding of DRP1 to actin filaments increases its GTPase activity, required for membrane constriction and fission [62]. To date, a number of different adaptor/receptor proteins promoting the recruitment of DRP1 to mitochondrial membranes have been characterized. However, the mitochondrial fission factor (MFF) [63] and mitochondrial dynamics proteins 49 and 51 (MiD49 and MiD51) [64] are the DRP1 adaptor proteins currently receiving the most attention. Several lines of evidence identified MFF as the primary adaptor protein for DRP1 recruitment at mitochondrial division sites. The silencing of *MFF* in HeLa cells resulted in reduced DRP1 aggregation on mitochondrial membranes, which was associated with fused and elongated mitochondria [63]. Conversely, MFF overexpression led to excessive mitochondrial fragmentation accompanied by increased DRP1 recruitment on mitochondrial fission sites [65]. Further to MFF, knockdown studies have identified the OMM proteins MiD49 and MiD51 as additional mediators of DRP1-recruitment at sites of mitochondrial fission. DRP1 expression levels and mitochondrial membrane targeting were found to be significantly reduced following MiD49 and MiD51 depletion, whereas MFF was still localized to mitochondrial fission sites [64,66]. The above suggest that MiD49 and MiD51 recruit DRP1 to the sites of mitochondrial constriction independently of MFF. On the contrary, conflicting evidence shows that the overexpression of MiD51 accelerates mitochondrial elongation rather than promoting mitochondrial fission and hence, the precise mechanism of DRP1 recruitment at mitochondrial fission sites is still not completely understood [67]. An additional dynamin-related protein, DNM2, was shown to play an important role in the physical separation of mitochondria following the recruitment and assembly of DRP1 at constriction sites. Knockdown of DNM2 resulted in an elongated mitochondrial network featuring constricted regions between preassembled DRP1 polymeric complexes [68]. On the other hand, contradictory data in mouse fibroblasts and HeLa cells lacking DNM2 revealed no significant changes in mitochondrial morphology and confirmed unperturbed fission rates, suggesting that DNM2 is dispensable for mitochondrial fission [52]. The mitochondrial fission 1 protein (FIS1) has been initially implicated in the recruitment of DNM1, the yeast homolog of DRP1, to the OMM through interaction with MDV1 and CAF4 [69]. Accordingly, the overexpression of human FIS1 (hFIS1) was found to induce mitochondrial fragmentation, whereas FIS1 depletion resulted in elongated and fused mitochondria [70,71,72,73]. However, the specific role of FIS1 in mitochondrial fission in mammals remains unknown because of the apparent absence of MDV1 and CAF4 homologs and reported contradictory findings. It has been recently demonstrated that mitochondrial fragmentation mediated by hFIS1 occurs independently of DRP1 and DNM2. hFIS1 was found to not actively promote mitochondrial fragmentation but instead to interact with MFN1, MFN2 and OPA1 and block their GTPase activity, thereby inhibiting mitochondrial fusion and shifting the balance of mitochondrial dynamics towards fission [72] (Figure 1).

Mitochondrial fission enables the separation of defective and depolarized mitochondria. It is thus of no surprise that mitochondrial fragmentation is often associated with conditions of cellular stress and mitochondrial dysfunction. Furthermore, a reciprocal relationship exists between mitochondrial fission and the process of mitophagy. In particular, mitochondrial fragmentation was found to be necessary for mitophagy since the inhibition of fission decreases mitophagy [74]. Conversely, key mitophagy players, such as the ubiquitin ligase Parkin, influence mitochondrial dynamics by targeting MFN1 and MFN2 for proteasomal degradation and thus promoting mitochondrial fission [75].

### 3.3. The Role of ER–Mitochondria Contact Sites in Mitochondrial Division

The observation that the diameter of DRP1 polymeric rings (~150 nm) is much smaller than the average diameter of mitochondrial tubules (~300 nm) led to the postulation of the existence of a “pre-DRP1 constriction” step that reduces mitochondrial tubule diameter [59]. As revealed by electron microscopy tomography and live-cell imaging, these preconstriction sites are marked by sites of contact between ER and mitochondria. In particular, the ER was shown to wrap around mitochondria at specific sites, facilitating constriction and demarcating the sites of mitochondrial fission (Figure 1). Importantly, these events also occur in cells in which DRP1 or its adaptor proteins MFF, MiD49 or MiD51 have been knocked down, suggesting that preconstriction at ER–mitochondria contact sites (ERMCS) occurs upstream of DRP1 assembly [76].

Further insights into the mechanism of mitochondrial constriction at ER–mitochondria contact sites and mitochondrial fission were provided by a study that identified the ER-associated form of inverted formin 2 (INF2) as an important player in this process. INF2 was found to act upstream of DRP1 and promote actin polymerization at ERMCS, providing the force to facilitate the initial mitochondrial constriction followed by DRP1 recruitment and assembly to complete the division process [77]. It has also been shown that a population of DRP1 oligomers is assembled on the ER and that ER-bound DRP1 can be subsequently transferred to mitochondria. The inhibition of actin polymerization eliminates DRP1 oligomers at the ER and reduces mitochondrial division, suggesting that the ER serves as the initial platform for DRP1 oligomerization and that actin polymerization is an essential prerequisite for mitochondrial division [78]. A further study demonstrated the involvement of myosin IIA and IIB in mitochondria tubule preconstriction and the recruitment of DRP1. This may suggest that the contraction of myosin II filaments could provide the necessary force to facilitate preconstriction by pulling mitochondrial membranes together [79]. In addition to the activation of DRP1 recruitment at sites of mitochondrial division, actin polymerization promoted by INF2 was shown to stimulate calcium influx into the mitochondrial matrix. Moreover, the above rise in mitochondrial-matrix calcium was found to promote IMM constriction in a DRP1-independent manner. Interestingly, IMM division occurs prior to the separation of the OMM. Collectively, the reported findings suggest a dual effect of INF2-mediated actin polymerization at sites of mitochondrial constriction: (a) the recruitment of DRP1 and (b) calcium influx into the matrix leading to OMM and IMM constriction, respectively [80].

In addition to their role in determining the sites of mitochondrial fission, ERMCS have been identified as important glucose-sensing hubs in tissues such as the liver and muscle [81]. The availability of nutrients impacts on ER–mitochondria communication also affecting mitochondrial dynamics. Although the precise effects of glucose on ER–mitochondrial tethering remain controversial, nutrient availability is associated with mitochondrial fragmentation, whereas nutrient restriction is associated with increased fusion [82]. The above glucose-sensing function of ERMCS and its effects on mitochondrial dynamics has important implications during nutritional transition and in pathological conditions of imbalanced glucose homeostasis. A protein potentially implicated in the glucose-sensing function of ERMCS is the glycogen-binding protein Stbd1. It has been reported that Stbd1 localizes to ERMCS and influences ER–mitochondrial tethering and mitochondrial dynamics. In particular, Stbd1 silencing was associated with reduced ER–mitochondrial contact and enhanced mitochondrial fusion, whereas its forced overexpression at ERMCS increased ER–mitochondrial tethering and resulted in mitochondrial fragmentation. Interestingly, Stbd1 is targeted to ERMCS when bound to glycogen and not in glycogen-free form, which may signal glucose availability to ERMCS [83]. Moreover, we have recently demonstrated that Stbd1 is a downstream target of the unfolded protein response pathway that is activated in conditions of ER stress [84]. Interestingly, ER stress is transiently induced in the liver upon refeeding [85], which may further support a role for Stbd1 in glucose sensing at ERMCS.

## 4. Mitochondrial Dynamics and Associated Diseases

Unequivocally, the steady-state and dynamic equilibrium of the mitochondrial network is critical for preserving optimal function at the organismal level. Disruption of the delicate equilibrium between the two opposing processes of fusion and fission results in a fragmented or elongated mitochondrial network that has been associated with several pathological conditions. Besides the mutations in nuclear or mitochondrially encoded genes that are associated with monogenic diseases of mitochondrial dysfunction [86], aberrations in mitochondrial structure have recently emerged as a pathogenic mechanism of more complex diseases and opened a new perspective in disease pathology (Table 1). Examples of such pathologies include various metabolic conditions, cancer and a broad spectrum of neurodegenerative diseases [87,88,89].

### 4.1. Defects in Mitochondrial Fusion Mediators and Disease

Systematic studies have shown that a plethora of human diseases are associated with excessive mitochondrial fragmentation as a result of defective fusion. 

Neurons are cells of extremely high energy demand and are thus particularly vulnerable to mitochondrial dysfunction. Alterations in the mitochondrial network and consequent mitochondrial dysfunction have been suggested to occur as prominent early features in neurodegenerative diseases [90]. Nowadays, there is an increasing number of neurodegenerative disorders that are associated with mutations in mitochondrial fusion genes. Genetic alterations in the *MFN2* gene represent the most common molecular cause of Charcot–Marie–Tooth disease Type 2A (CMT2A), a dominantly inherited form of peripheral neuropathy. To date, more than 100 different mutations affecting *MFN2* have been reported in patients with CMT2A (available from: uantwerpen.vib.be). The typical clinical symptoms of CMT2A involve the progressive atrophy of distal limb muscles that usually leads to wheelchair dependency in patients and a decrease of deep-tendon reflexes linked to foot deformities and distal sensory loss [17]. Structural studies have shown that the majority of CMT2A-associated mutations in *MFN2* are mapped to four distinct functional zones and are mostly associated with severe CMT2A phenotypes, whereas mutations beyond the functional domains lead to milder symptoms. At the molecular level, MFN2 mutants encoding truncated or dimerization-incompetent proteins are more likely to cause disease due to decreased levels of normal MFN2, whereas mutants capable of dimerization and fusion tend to be pathogenic by hijacking normal MFN1 and MFN2 activity [91]. In a recent study, the pharmacological activation of MFN2 using mitofusin agonists was shown to overcome fusion suppression and reversed mitochondrial defects in cultured neurons expressing CMT2A mutants [92]. Moreover, overexpression of MFN1 in a CMT2A mouse model expressing mutant MFN2 was shown to reverse mitochondrial network defects and mitigate phenotypic abnormalities, supporting the notion that imbalances in mitofusin function is a key determinant in disease development [93]. *MFN2* mutations have been also linked to another type of CMT disease, designated as hereditary motor and sensory neuropathy (HMSN) Type IV, which is characterized by subacute optic neuropathy, bilateral visual impairment and color-vision defects. Interestingly, mutations in the IMM fusion mediator OPA1 underlie the most prevalent form of autosomal dominant optic neuropathy, suggesting a functional overlap of OPA1 with MFN2. The above further implies that balanced mitochondrial dynamics are of particular importance for the proper function of the optic nerve [94,95,96]. 

Given their key regulatory role in energy metabolism, mitochondria respond to the availability of nutrients and energy demands by adjusting mitochondrial dynamics to maintain homeostasis. As mentioned earlier, under conditions of nutrient shortage and increased energy demand, the mitochondrial network appears elongated, whereas ample nutrient supply and decreased energy demand are associated with mitochondrial fragmentation [97,98]. It is therefore not surprising that imbalances in mitochondrial dynamics have been recognized as central players in the pathophysiology of obesity and diabetes. Ultrastructural observations in skeletal muscle of obese and type-2 diabetic subjects revealed perturbed structural organization of the mitochondrial network, characterized by small and fragmented mitochondria as compared to healthy controls [99]. Altered expression of MFN1 and MFN2 has been implicated by several studies for abnormal mitochondrial metabolism and the development of diabetes. A study examining MFN2 expression levels in the skeletal muscle of obese and diabetic patients showed reduced MFN2 expression and decreased insulin sensitivity associated with alterations in mitochondrial morphology and aberrant muscle metabolism [100]. In line with the above, a different study reported reduced mRNA levels and protein expression of MFN2 in the skeletal muscle of obese subjects and high-fat Zucker rats, while subsequent electron microscopy analysis revealed disturbed architecture and fragmentation of the mitochondrial network. In addition, the partial abolishment of MFN2 expression led to abnormal mitochondrial metabolism in vitro [101], which is a key risk factor in the development of diabetes. Additional observations in a mouse model of high-fat-diet-induced obesity associated with mitochondrial dysfunction showed that MFN1 and MFN2 expression in skeletal muscle was significantly decreased, concomitant with an increase in the expression of the mitochondrial fission mediators DRP1 and FIS1, thereby shifting mitochondrial dynamics towards fission [102]. Corroborating data from another in vivo study demonstrated that mice lacking MFN1 in proopiomelanocortin (POMC) neurons exhibit abnormal glucose levels due to perturbations in insulin secretion, unveiling a new role for mitochondrial dynamics in the regulation of insulin signaling and glucose homeostasis [103]. 

As mentioned earlier, OPA1 mutants are responsible for the autosomal dominant optic atrophy-1 (ADOA), a neuro-ophthalmic condition primarily characterized by impairment of visual acuity and generalized color-vision deficits [18,104]. Besides ocular symptoms, a large number of OPA1 variants have been associated with additional clinical complications including auditory neuropathy, myopathy and ataxia, which define a new type of disease, termed ADOA-plus syndrome [105]. Since the precise pathomechanism of ADOA is not yet fully clear and given the large number of OPA1 mutants that result in truncated products, it has been suggested that haploinsufficiency may represent one of the major disease-causing mechanisms [106]. Certainly, the broad mutation spectrum and the difficulty to establish phenotype–genotype correlations in ADOA patients support the notion that there are additional genetic factors implicated in disease development that have not yet been elucidated. Regardless of the underlying mechanism, mitochondrial fragmentation has been described as a common feature in ADOA with disease severity being proportional to the extent of fragmentation, underscoring the involvement of defective mitochondrial fusion in disease development [107,108]. Rare OPA1 mutants have been also reported in patients characterized by parkinsonism and dementia, conferring further evidence of the involvement of abnormal mitochondrial fusion in the pathogenesis of other common neurodegenerative diseases [109,110].

### 4.2. Defects in Mitochondrial Fission Mediators and Disease

In addition to aberrations in the function of mitochondrial fusion mediators, perturbations of the mitochondrial fission machinery have received special attention with regards to their relevance in the development and progression of human pathologies.

Disturbances in mitochondrial fission and in particular in the function of the DRP1 protein have been proposed to play a principal role in the initiation and progression of cancer, though different cancers are known to have distinct oncogenic backgrounds and etiologies. It has been shown that lung-cancer cell lines and lung adenocarcinoma cells from patients exhibit excessive mitochondrial network fragmentation as a result of increased DRP1 expression and reduced MFN2 levels [111]. Moreover, evidence from the same study revealed that the DRP1 protein was not only overexpressed across two different adenocarcinoma cell lines but that its activity was further enhanced after phosphorylation on Ser-616. In contrast, DRP1 inhibition or MFN2 overexpression resulted in reduced proliferation of cancer cells, increased apoptosis and significant regression of tumor growth in vivo [111]. In a second study, DRP1 and mitochondrial dynamics were shown to have a principal role in brain-tumor development. Brain tumor initiating cells (BTICs), which represent a distinguished subpopulation of tumor cells, displayed an increased rate of small and fragmented mitochondria and DRP1 hyperactivation, as compared to non-BTICs. Selective inhibition of DRP1 eliminated tumor growth and increased tumor latency and survival in vivo [112]. Furthermore, a *DRP1*-based large-scale analysis of cancer genomes across various cancer types revealed a robust association of *DRP1* with cell-cycle genes, postulating a role for DRP1 and mitochondrial fission in the regulation of cell proliferation [113]. It is not yet clear whether mitochondrial fragmentation triggers the transformation of cancerous cells, promotes cell-cycle progression or changes the susceptibility of tumor cells to apoptosis, but certainly mitochondrial division is evidently involved in human tumorigenesis.

Over the last few years, mitochondrial fission has been increasingly implicated in a number of neurodegenerative disorders, including Alzheimer’s disease (AD), the leading cause of senile and presenile dementia. Pathologically, AD is characterized by generalized cortical atrophy and aggregations of neurofibrillary tangles and amyloid plaques, the density of which appears to correlate with the clinical presentation [114]. Expression analysis of the primary fusion and fission mediators in postmortem AD brains revealed abnormal levels of DRP1, MFN1, MFN2, FIS1 and OPA1, suggesting that altered equilibrium of mitochondrial dynamics may represent a mechanism underlying neuronal dysfunction in AD [115]. AD-associated Aβ plaques’ deposition and cognitive impairment is ameliorated upon DRP1 inhibition, as reported by recent studies [116,117]. The selective inhibition of DRP1-mediated mitochondrial division using mitochondrial-division inhibitor 1 (Mdivi-1) was shown to improve synaptic damage and mitochondrial function due to diminished mitochondrial fission in AD neurons [116]. In a second study, the selective inhibition of DRP1 by Mdivi-1 in Aβ-treated neurons blocked mitochondrial fragmentation and improved mitochondrial function. Furthermore, DRP1 inhibition led to a significant decrease of Aβ plaques in the brain of an AD mouse model and alleviated cognitive symptoms [117]. More recent evidence from fibroblasts derived from AD patients showed increased interaction of DRP1 with its adaptor protein, FIS1, resulting in excessive mitochondrial fission and dysfunction. Conversely, pharmacological inhibition of DRP1/FIS1 interaction improved mitochondrial function in cultured neurons and significantly mitigated the pathological features in the brain of an AD mouse model [118]. In line with the above, a spectrum of mitochondrial abnormalities, including functional distress and the accumulation of fragmented mitochondria, were identified in close proximity to areas with dense amyloid plaque formation in the brains of transgenic animals with AD pathology [119].

Besides AD, mitochondrial dysfunction in neuronal cells is also regarded as one of the main mechanisms for the development of Parkinson’s disease (PD), a degenerative disorder of the central nervous system affecting the motor activity of millions of people worldwide. The histopathological hallmark of PD is the intraneuronal build-up of misfolded proteins such as a-synuclein in the substantia nigra leading to the loss of dopaminergic neurons and concomitant dopamine deficiency [120]. Despite the so far obscure etiology of PD, multiple lines of evidence propose mitochondrial dysfunction as an underlying mechanism with potential deleterious effects on neuronal activity. The discovery of multiple mutations in the mitochondrial quality-control protein PTEN-induced putative kinase 1 (PINK1), which are responsible for the autosomal recessive familial PD [121], and, given the implication of PINK1 in mitochondrial dynamics [122,123], the conclusion is that mitochondrial integrity in PD pathogenesis is of central importance. Indeed, it has been demonstrated that the depletion of PINK1 or expression of a PINK1 mutant protein heavily tipped the balance towards fission and excessive mitochondrial fragmentation, while overexpression of the wild-type protein promoted fusion by increasing MFN2 expression [124]. Corroborating research indicated that PINK1 deficiency in a human dopaminergic cell line leads to increased mitochondrial fragmentation and increased mitochondrial autophagy, resulting in “autophagic stress”, a critical determinant of neurodegeneration [123]. Consistent with the previous studies, abnormal mitochondrial fission has been further documented as a key factor in the pathogenic pathway of PD by a different study, which reported increased expression of DRP1 and excessive mitochondrial fragmentation in a cellular model of toxin-induced PD [125]. Additional evidence from in vivo models characterized by mitochondrial dysfunction in the nigrostriatal system suggest that the inhibition of mitochondrial fission by the blocking of DRP1 function rescues dopamine-release deficits and attenuates dopaminergic neurotoxicity, thus providing a neuroprotective effect in the nigrostriatal pathway [126]. Furthermore, more recent data from in vitro and in vivo models suggest that sporadic PD is attributed to excessive mitochondrial fragmentation triggered by elevated levels of DRP1 [127]. Accordingly, inhibition of DRP1 hyperactivation in a mouse model of PD attenuated dopaminergic neuronal loss due to reduced mitochondrial translocation of DRP1 and other proteins involved in programmed cell death [128].

**Table 1:** Major mitochondrial fusion and fission effectors in mammals and associated diseases. MFN1/MFN2, mitofusin-1 and -2; OMM, outer mitochondrial membrane; IMM, inner mitochondrial membrane; ER, endoplasmic reticulum; CMT2A, Charcot–Marie–Tooth type 2A; HMSNs, hereditary motor and sensory neuropathies; OPA1, optic atrophy type 1; DRP1, dynamin-related protein-1; MFF, mitochondrial fission factor; FIS1, fission mitochondrial 1; MIEF1 /MIEF2, mitochondrial elongation factor 1 and 2; DNM2, dynamin 2; CNM1, centronuclear myopathy 1; DI-CMTB, dominant intermediate Charcot–Marie–Tooth disease type B; CMT2M, Charcot–Marie–Tooth disease axonal type 2M.

Gene Symbol	Protein Localization	Proposed Function	Most Common Pathologies Related to Gene Mutation	Cellular Effect	References
***MFN2***	OMM and ER	Mediator of mitochondrial tethering and OMM fusion	CMT2A and other HMSNs	Altered mitochondrial distribution/Reduced ER–mitochondria tethering	[17,94,129,130]
***MFN1***	OMM	Mediator of mitochondrial tethering and OMM fusion	Not known		[42]
***OPA1***	IMM	Mediator of IMM fusion	Optic atrophy 1 Mitochondrial DNA depletion syndrome Parkinsonism and dementia	Incomplete mitochondrial fusion/fragmented mitochondria	[18,109,131]
***DRP1***	Cytosol & OMM	Mediator of mitochondrial fission	Encephalopathy Optic atrophy	Excessive mitochondrial fusion	[20,21,132]
***MFF***	OMM	DRP1 recruitment protein/ fission accessory protein	Encephalopathy Optic atrophy Peripheral neuropathy	Defective fission/tubular mitochondria	[133,134]
***FIS1***	OMM	DRP1 recruitment protein/ fission accessory protein	Not known		[135]
***MIEF2/MiD49***	OMM	DRP1 recruitment protein/ fission accessory protein	Mitochondrial myopathy	Elongated mitochondria/higher frequency of fusion events	[136]
***MIEF1/MiD51***	OMM	DRP1 recruitment protein/ fission accessory protein	Optic neuropathy	Disrupted mitochondrial fission/fusion dynamics	[137]
***DNM2***	Cytosol & OMM	Mediator of mitochondrial fission	CNM1, DI-CMTB and CMT2M	Disrupted actin filaments and microtubule network	[138,139,140]

## 5. Concluding Remarks

Mitochondrial fusion and fission fine-tune cardinal processes of cellular life, and cumulative evidence has attracted remarkable interest into the role of mitochondrial remodeling in biological function. Undoubtedly, the scientific conception of mitochondrial dynamics and a comprehensive understanding of the mechanisms controlling mitochondrial architecture have progressed considerably in the last years. Of course, there are still many unresolved questions and additional proteins implicated in mitochondrial dynamics that remain to be identified. Nonetheless, the association between imbalances in mitochondrial fusion and fission with a steadily increasing number of pathological conditions and the provided proof-of-concept that restoration of mitochondrial network structure ameliorates disease renders mitochondrial dynamics as a major culprit for disease pathogenesis and an attractive target for therapy.

## Data Availability

Not applicable.

## References

[1] Palade G.E. (1953). AN electron microscope study of the mitochondrial structure. J. Histochem. Cytochem..

[2] Roger A.J., Muñoz-Gómez S.A., Kamikawa R. (2017). The Origin and Diversification of Mitochondria. Curr. Biol..

[3] Vakifahmetoglu-Norberg H., Ouchida A.T., Norberg E. (2017). The role of mitochondria in metabolism and cell death. Biochem. Biophys. Res. Commun..

[4] Ramos E.S., Larsson N.-G., Mourier A. (2016). Bioenergetic roles of mitochondrial fusion. Biochim. Biophys. Acta (BBA) Bioenerg..

[5] Benda C. (1898). Ueber die spermatogenese der vertebraten und höherer evertebraten, II. Theil: Die histiogenese der spermien. Arch. Anat. Physiol..

[6] Gustafsson C.M., Falkenberg M., Larsson N.-G. (2016). Maintenance and Expression of Mammalian Mitochondrial DNA. Annu. Rev. Biochem..

[7] Kühlbrandt W. (2015). Structure and function of mitochondrial membrane protein complexes. BMC Biol..

[8] Wai T., Langer T. (2016). Mitochondrial Dynamics and Metabolic Regulation. Trends Endocrinol. Metab..

[9] Trotta A.P., Chipuk J.E. (2017). Mitochondrial dynamics as regulators of cancer biology. Cell. Mol. Life Sci..

[10] Pernas L., Scorrano L. (2016). Mito-Morphosis: Mitochondrial Fusion, Fission, and Cristae Remodeling as Key Mediators of Cellular Function. Annu. Rev. Physiol..

[11] Rodolfo C., Campello S., Cecconi F. (2018). Mitophagy in neurodegenerative diseases. Neurochem. Int..

[12] Gustafsson Å.B., Dorn G.W. (2019). Evolving and Expanding the Roles of Mitophagy as a Homeostatic and Pathogenic Process. Physiol. Rev..

[13] Hoppins S., Lackner L., Nunnari J. (2007). The Machines that Divide and Fuse Mitochondria. Annu. Rev. Biochem..

[14] Chen H., Detmer S.A., Ewald A.J., Griffin E.E., Fraser S.E., Chan D.C. (2003). Mitofusins Mfn1 and Mfn2 coordinately regulate mitochondrial fusion and are essential for embryonic development. J. Cell Biol..

[15] Santel A., Fuller M.T. (2001). Control of mitochondrial morphology by a human mitofusin. J. Cell Sci..

[16] Smirnova E., Griparic L., Shurland D.-L., Van Der Bliek A.M. (2001). Dynamin-related Protein Drp1 Is Required for Mitochondrial Division in Mammalian Cells. Mol. Biol. Cell.

[17] Zuchner S., Mersiyanova I.V., Muglia M., Bissar-Tadmouri N., Rochelle J., Dadali E.L., Vance J.M. (2004). Mutations in the mitochondrial GTPase mitofusin 2 cause Charcot-Marie-Tooth neuropathy type 2A. Nat. Genet..

[18] Delettre C., Lenaers G., Griffoin J.-M., Gigarel N., Lorenzo C., Belenguer P., Pelloquin L., Grosgeorge J., Turc-Carel C., Perret E. (2000). Nuclear gene OPA1, encoding a mitochondrial dynamin-related protein, is mutated in dominant optic atrophy. Nat. Genet..

[19] Yoon G., Malam Z., Paton T., Marshall C.R., Hyatt E., Ivakine Z., Scherer S.W., Lee K.-S., Hawkins C., Cohn R.D. (2016). Lethal Disorder of Mitochondrial Fission Caused by Mutations in DNM1L. J. Pediatr..

[20] Batzir N.A., Bhagwat P.K., Eble T.N., Liu P., Eng C.M., Elsea S.H., Robak L.A., Scaglia F., Goldman A.M., Dhar S.U. (2019). De novo missense variant in the GTPase effector domain (GED) of DNM1L leads to static encephalopathy and seizures. Mol. Case Stud..

[21] Fahrner J.A., Liu R., Perry M.S., Klein J., Chan D.C. (2016). A novel de novo dominant negative mutation in DNM1L impairs mitochondrial fission and presents as childhood epileptic encephalopathy. Am. J. Med Genet. Part A.

[22] Mishra P. (2016). Interfaces between mitochondrial dynamics and disease. Cell Calcium.

[23] Chan D.C. (2020). Mitochondrial Dynamics and Its Involvement in Disease. Annu. Rev. Pathol. Mech. Dis..

[24] Giacomello M., Pyakurel A., Glytsou C., Scorrano L. (2020). The cell biology of mitochondrial membrane dynamics. Nat. Rev. Mol. Cell Biol..

[25] Malka F., Guillery O., Cifuentes-Diaz C., Guillou E., Belenguer P., Lombès A., Rojo M. (2005). Separate fusion of outer and inner mitochondrial membranes. EMBO Rep..

[26] Saxton W.M., Hollenbeck P.J. (2012). The axonal transport of mitochondria. J. Cell Sci..

[27] Chen H., Chan D.C. (2010). Physiological functions of mitochondrial fusion. Ann. N. Y. Acad. Sci..

[28] Hales K.G., Fuller M.T. (1997). Developmentally Regulated Mitochondrial Fusion Mediated by a Conserved, Novel, Predicted GTPase. Cell.

[29] Cohen M.M., Tareste D. (2018). Recent insights into the structure and function of Mitofusins in mitochondrial fusion. F1000Research.

[30] De Brito O.M., Scorrano L. (2008). Mitofusin 2 tethers endoplasmic reticulum to mitochondria. Nature.

[31] Chen H., Chomyn A., Chan D.C. (2005). Disruption of Fusion Results in Mitochondrial Heterogeneity and Dysfunction. J. Biol. Chem..

[32] Shepard K.A., Yaffe M.P. (1999). The Yeast Dynamin-like Protein, Mgm1p, Functions on the Mitochondrial Outer Membrane to Mediate Mitochondrial Inheritance. J. Cell Biol..

[33] Pelloquin L., Belenguer P., Menon Y., Gas N., Ducommun B. (1999). Fission yeast Msp1 is a mitochondrial dynamin-related protein. J. Cell Sci..

[34] Olichon A., Emorine L.J., Descoins E., Pelloquin L., Brichese L., Gas N., Guillou E., Delettre C., Valette A., Hamel C.P. (2002). The human dynamin-related protein OPA1 is anchored to the mitochondrial inner membrane facing the inter-membrane space. FEBS Lett..

[35] Wong E.D., Wagner J.A., Gorsich S.W., McCaffery J.M., Shaw J.M., Nunnari J. (2000). The Dynamin-Related Gtpase, Mgm1p, Is an Intermembrane Space Protein Required for Maintenance of Fusion Competent Mitochondria. J. Cell Biol..

[36] Satoh M., Hamamoto T., Seo N., Kagawa Y., Endo H. (2003). Differential sublocalization of the dynamin-related protein OPA1 isoforms in mitochondria. Biochem. Biophys. Res. Commun..

[37] Griparic L., van der Wel N.N., Orozco I.J., Peters P.J., van der Bliek A.M. (2004). Loss of the Intermembrane Space Protein Mgm1/OPA1 Induces Swelling and Localized Constrictions along the Lengths of Mitochondria. J. Biol. Chem..

[38] Cipolat S., De Brito O.M., Zilio B.D., Scorrano L. (2004). OPA1 requires mitofusin 1 to promote mitochondrial fusion. Proc. Natl. Acad. Sci. USA.

[39] Li D., Wang J., Jin Z., Zhang Z. (2019). Structural and evolutionary characteristics of dynamin-related GTPase OPA1. PeerJ.

[40] Whyte J.R.C., Munro S. (2002). Vesicle tethering complexes in membrane traffic. J. Cell Sci..

[41] Koshiba T. (2004). Structural Basis of Mitochondrial Tethering by Mitofusin Complexes. Science.

[42] Cao Y.-L., Meng S., Chen Y., Feng J.-X., Gu D.-D., Yu B., Li Y.-J., Yang J.-Y., Liao S., Chan D.C. (2017). MFN1 structures reveal nucleotide-triggered dimerization critical for mitochondrial fusion. Nat. Cell Biol..

[43] Song Z., Chen H., Fiket M., Alexander C., Chan D.C. (2007). OPA1 processing controls mitochondrial fusion and is regulated by mRNA splicing, membrane potential, and Yme1L. J. Cell Biol..

[44] Griparic L., Kanazawa T., Van Der Bliek A.M. (2007). Regulation of the mitochondrial dynamin-like protein Opa1 by proteolytic cleavage. J. Cell Biol..

[45] Duvezin-Caubet S., Jagasia R., Wagener J., Hofmann S., Trifunovic A., Hansson A., Chomyn A., Bauer M.F., Attardi G., Larsson N.-G. (2006). Proteolytic Processing of OPA1 Links Mitochondrial Dysfunction to Alterations in Mitochondrial Morphology. J. Biol. Chem..

[46] Vogel F., Herlan M., Bornhövd C., Neupert W., Reichert A.S. (2003). Processing of Mgm1 by the Rhomboid-type Protease Pcp1 Is Required for Maintenance of Mitochondrial Morphology and of Mitochondrial DNA. J. Biol. Chem..

[47] DeVay R.M., Dominguez-Ramirez L., Lackner L.L., Hoppins S., Stahlberg H., Nunnari J. (2009). Coassembly of Mgm1 isoforms requires cardiolipin and mediates mitochondrial inner membrane fusion. J. Cell Biol..

[48] Ge Y., Shi X., Boopathy S., McDonald J., Smith A.W., Chao L.H. (2020). Two forms of Opa1 cooperate to complete fusion of the mitochondrial inner-membrane. eLife.

[49] Ban T., Ishihara T., Kohno H., Saita S., Ichimura A., Maenaka K., Oka T., Mihara K., Ishihara N. (2017). Molecular basis of selective mitochondrial fusion by heterotypic action between OPA1 and cardiolipin. Nat. Cell Biol..

[50] Bleazard W., McCaffery J.M., King E.J., Bale S., Mozdy A., Tieu Q., Nunnari J., Shaw J.M. (1999). The dynamin-related GTPase Dnm1 regulates mitochondrial fission in yeast. Nat. Cell Biol..

[51] Tieu Q., Nunnari J. (2000). Mdv1p Is a Wd Repeat Protein That Interacts with the Dynamin-Related Gtpase, Dnm1p, to Trigger Mitochondrial Division. J. Cell Biol..

[52] Fonseca T.B., Sánchez-Guerrero Á., Milosevic I., Raimundo N. (2019). Mitochondrial fission requires DRP1 but not dynamins. Nat. Cell Biol..

[53] Francy C.A., Alvarez F.J.D., Zhou L., Ramachandran R., Mears J.A. (2015). The Mechanoenzymatic Core of Dynamin-related Protein 1 Comprises the Minimal Machinery Required for Membrane Constriction. J. Biol. Chem..

[54] Whitley B.N., Lam C., Cui H., Haude K., Bai R., Escobar L., Hamilton A., Brady L., Tarnopolsky M.A., Dengle L. (2018). Aberrant Drp1-mediated mitochondrial division presents in humans with variable outcomes. Hum. Mol. Genet..

[55] Fröhlich C., Grabiger S., Schwefel D., Faelber K., Rosenbaum E., Mears J., Rocks O., Daumke O. (2013). Structural insights into oligomerization and mitochondrial remodelling of dynamin 1-like protein. EMBO J..

[56] Kalia R., Wang R.Y.-R., Yusuf A., Thomas P.V., Agard D.A., Shaw J.M., Frost A. (2018). Structural basis of mitochondrial receptor binding and constriction by DRP1. Nat. Cell Biol..

[57] Otera H., Mihara K. (2011). Discovery of the membrane receptor for mitochondrial fission GTPase Drp1. Small GTPases.

[58] Lackner L.L., Horner J.S., Nunnari J. (2009). Mechanistic Analysis of a Dynamin Effector. Science.

[59] Mears J.A., Lackner L.L., Fang S., Ingerman E., Nunnari J., Hinshaw J.E. (2010). Conformational changes in Dnm1 support a contractile mechanism for mitochondrial fission. Nat. Struct. Mol. Biol..

[60] Otera H., Ishihara N., Mihara K. (2013). New insights into the function and regulation of mitochondrial fission. Biochim. Biophys. Acta (BBA) Bioenerg..

[61] Elgass K.D., Smith E.A., Legros M.A., Larabell C.A., Ryan M.T. (2015). Analysis of ER-mitochondria contacts using correlative fluorescence microscopy and soft X-ray tomography of mammalian cells. J. Cell Sci..

[62] Ji W.-K., Hatch A.L., Merrill R.A., Strack S., Higgs H.N. (2015). Actin filaments target the oligomeric maturation of the dynamin GTPase Drp1 to mitochondrial fission sites. eLife.

[63] Gandre-Babbe S., van der Bliek A.M. (2008). The novel tail-anchored membrane protein Mff controls mitochondrial and peroxisomal fission in mammalian cells. Mol. Biol. Cell.

[64] Palmer C.S., Osellame L.D., Laine D., Koutsopoulos O.S., Frazier A.E., Ryan M.T. (2011). MiD49 and MiD51, new components of the mitochondrial fission machinery. EMBO Rep..

[65] Otera H., Wang C., Cleland M.M., Setoguchi K., Yokota S., Youle R.J., Mihara K. (2010). Mff is an essential factor for mitochondrial recruitment of Drp1 during mitochondrial fission in mammalian cells. J. Cell Biol..

[66] Losón O.C., Song Z., Chen H., Chan D.C. (2013). Fis1, Mff, MiD49, and MiD51 mediate Drp1 recruitment in mitochondrial fission. Mol. Biol. Cell.

[67] Zhao J., Liu T., Jin S., Wang X., Qu M., Uhlén P., Tomilin N., Shupliakov O., Lendahl U., Nistér M. (2011). Human MIEF1 recruits Drp1 to mitochondrial outer membranes and promotes mitochondrial fusion rather than fission. EMBO J..

[68] Lee J.E., Westrate L.M., Wu H., Page C., Voeltz G.K. (2016). Multiple dynamin family members collaborate to drive mitochondrial division. Nat. Cell Biol..

[69] Zhang Y., Chan D.C. (2007). Structural basis for recruitment of mitochondrial fission complexes by Fis1. Proc. Natl. Acad. Sci. USA.

[70] Yoon Y., Krueger E.W., Oswald B.J., McNiven M.A. (2003). The Mitochondrial Protein hFis1 Regulates Mitochondrial Fission in Mammalian Cells through an Interaction with the Dynamin-Like Protein DLP1. Mol. Cell. Biol..

[71] Koch A., Yoon Y., Bonekamp N.A., McNiven M.A., Schrader M. (2005). A Role for Fis1 in Both Mitochondrial and Peroxisomal Fission in Mammalian Cells. Mol. Biol. Cell.

[72] Yu R., Jin S., Lendahl U., Nistér M., Zhao J. (2019). Human Fis1 regulates mitochondrial dynamics through inhibition of the fusion machinery. EMBO J..

[73] Stojanovski D., Koutsopoulos O.S., Okamoto K., Ryan M.T. (2004). Levels of human Fis1 at the mitochondrial outer membrane regulate mitochondrial morphology. J. Cell Sci..

[74] Twig G., Elorza A., Molina A.A.J., Mohamed H., Wikstrom J.D., Walzer G., Stiles L., Haigh S.E., Katz S., Las G. (2008). Fission and selective fusion govern mitochondrial segregation and elimination by autophagy. EMBO J..

[75] Tanaka A., Cleland M.M., Xu S., Narendra D.P., Suen D.-F., Karbowski M., Youle R.J. (2010). Proteasome and p97 mediate mitophagy and degradation of mitofusins induced by Parkin. J. Cell Biol..

[76] Friedman J.R., Lackner L.L., West M., DiBenedetto J.R., Nunnari J., Voeltz G.K. (2011). ER Tubules Mark Sites of Mitochondrial Division. Science.

[77] Korobova F., Ramabhadran V., Higgs H.N. (2013). An Actin-Dependent Step in Mitochondrial Fission Mediated by the ER-Associated Formin INF2. Science.

[78] Ji W.-K., Chakrabarti R., Stefan S., Schoenfeld L., Strack S., Higgs H.N. (2017). Receptor-mediated Drp1 oligomerization on endoplasmic reticulum. J. Cell Biol..

[79] Korobova F., Gauvin T.J., Higgs H.N. (2014). A Role for Myosin II in Mammalian Mitochondrial Fission. Curr. Biol..

[80] Chakrabarti R., Ji W.-K., Stan R.V., Sanz J.D.J., Ryan T.A., Higgs H.N. (2017). INF2-mediated actin polymerization at the ER stimulates mitochondrial calcium uptake, inner membrane constriction, and division. J. Cell Biol..

[81] Rieusset J. (2018). The role of endoplasmic reticulum-mitochondria contact sites in the control of glucose homeostasis: An update. Cell Death Dis..

[82] Theurey P., Rieusset J. (2017). Mitochondria-Associated Membranes Response to Nutrient Availability and Role in Metabolic Diseases. Trends Endocrinol. Metab..

[83] Demetriadou A., Morales-Sanfrutos J., Nearchou M., Baba O., Kyriacou K., Tate E.W., Drousiotou A., Petrou P.P. (2017). Mouse Stbd1 isN-myristoylated and affects ER–mitochondria association and mitochondrial morphology. J. Cell Sci..

[84] Lytridou A.A., Demetriadou A., Christou M., Potamiti L., Mastroyiannopoulos N.P., Kyriacou K., Phylactou L.A., Drousiotou A., Petrou P.P. (2020). Stbd1 promotes glycogen clustering during endoplasmic reticulum stress and supports survival of mouse myoblasts. J. Cell Sci..

[85] Sasako T., Ohsugi M., Kubota N., Itoh S., Okazaki Y., Terai A., Kubota T., Yamashita S., Nakatsukasa K., Kamura T. (2019). Hepatic Sdf2l1 controls feeding-induced ER stress and regulates metabolism. Nat. Commun..

[86] Alston C.L., Rocha M.C., Lax N.Z., Turnbull D.M., Taylor R.W. (2017). The genetics and pathology of mitochondrial disease. J. Pathol..

[87] Maycotte P., Marín-Hernández A., Goyri-Aguirre M., Anaya-Ruiz M., Reyes-Leyva J., Cortés-Hernández P. (2017). Mitochondrial dynamics and cancer. Tumor Biol..

[88] Gao J., Wang L., Liu J., Xie F., Su B., Wang X. (2017). Abnormalities of Mitochondrial Dynamics in Neurodegenerative Diseases. Antioxidants.

[89] Rovira-Llopis S., Bañuls C., Diaz-Morales N., Hernandez-Mijares A., Rocha M., Victor V.M. (2017). Mitochondrial dynamics in type 2 diabetes: Pathophysiological implications. Redox Biol..

[90] Zhu T., Chen J.-L., Wang Q., Shao W., Qi B. (2018). Modulation of Mitochondrial Dynamics in Neurodegenerative Diseases: An Insight Into Prion Diseases. Front. Aging Neurosci..

[91] Li Y.-J., Cao Y.-L., Feng J.-X., Qi Y., Meng S., Yang J.-F., Zhong Y.-T., Kang S., Chen X., Lan L. (2019). Structural insights of human mitofusin-2 into mitochondrial fusion and CMT2A onset. Nat. Commun..

[92] Rocha A.G., Franco A., Krezel A.M., Rumsey J.M., Alberti J.M., Knight W.C., Biris N., Zacharioudakis E., Janetka J.W., Baloh R.H. (2018). MFN2 agonists reverse mitochondrial defects in preclinical models of Charcot-Marie-Tooth disease type 2A. Science.

[93] Zhou Y., Carmona S., Muhammad A., Bell S., Landeros J., Vazquez M., Ho R., Franco A., Lu B., Dorn G.W. (2019). Restoring mitofusin balance prevents axonal degeneration in a Charcot-Marie-Tooth type 2A model. J. Clin. Investig..

[94] Züchner S., De Jonghe P., Jordanova A., Claeys K.G., Guergueltcheva V., Cherninkova S., Hamilton S.R., Van Stavern G., Krajewski K.M., Stajich J. (2006). Axonal neuropathy with optic atrophy is caused by mutations in mitofusin 2. Ann. Neurol..

[95] Leonardi L., Marcotulli C., Storti E., Tessa A., Serrao M., Parisi V., Santorelli F.M., Pierelli F., Casali C. (2015). Acute optic neuropathy associated with a novel MFN2 mutation. J. Neurol..

[96] Guerriero S., D’Oria F., Rossetti G., Favale R.A., Zoccolella S., Alessio G., Petruzzella V. (2020). CMT2A Harboring Mitofusin 2 Mutation with Optic Nerve Atrophy and Normal Visual Acuity. Int. Med. Case Rep. J..

[97] Molina A.J., Wikstrom J.D., Stiles L., Las G., Mohamed H., Elorza A., Shirihai O.S. (2009). Mitochondrial networking protects beta-cells from nutrient-induced apoptosis. Diabetes.

[98] Gomes L.C., Di Benedetto G., Scorrano L. (2011). During autophagy mitochondria elongate, are spared from degradation and sustain cell viability. Nat. Cell Biol..

[99] Kelley D.E., He J., Menshikova E.V., Ritov V.B. (2002). Dysfunction of Mitochondria in Human Skeletal Muscle in Type 2 Diabetes. Diabetes.

[100] Bach D., Naon D., Pich S., Soriano F.X., Vega N., Rieusset J., Laville M., Guillet C., Boirie Y., Wallberg-Henriksson H. (2005). Expression of Mfn2, the Charcot-Marie-Tooth Neuropathy Type 2A Gene, in Human Skeletal Muscle: Effects of Type 2 Diabetes, Obesity, Weight Loss, and the Regulatory Role of Tumor Necrosis Factor and Interleukin-6. Diabetes.

[101] Bach D., Pich S., Soriano F.X., Vega N., Baumgartner B., Oriola J., Daugaard J.R., Lloberas J., Camps M., Zierath J.R. (2003). Mitofusin-2 Determines Mitochondrial Network Architecture and Mitochondrial Metabolism. J. Biol. Chem..

[102] Liu R., Jin P., Yu L., Wang Y., Han L., Shi T., Li X. (2014). Impaired Mitochondrial Dynamics and Bioenergetics in Diabetic Skeletal Muscle. PLoS ONE.

[103] Ramírez S., Gómez-Valadés A.G., Schneeberger M., Varela L., Haddad-Tóvolli R., Altirriba J., Noguera E., Drougard A., Flores-Martínez Á., Imbernón M. (2017). Mitochondrial Dynamics Mediated by Mitofusin 1 Is Required for POMC Neuron Glucose-Sensing and Insulin Release Control. Cell Metab..

[104] Alexander C., Votruba M., Pesch U.E., Thiselton D.L., Mayer S., Moore A., Rodriguez M., Kellner U., Leo-Kottler B., Auburger G. (2000). OPA1, encoding a dynamin-related GTPase, is mutated in autosomal dominant optic atrophy linked to chromosome 3q28. Nat. Genet..

[105] Yu-Wai-Man P., Griffiths P.G., Gorman G.S., Lourenco C.M., Wright A.F., Auer-Grumbach M., Toscano A., Musumeci O., Valentino M.L., Caporali L. (2010). Multi-system neurological disease is common in patients with OPA1 mutations. Brain.

[106] Pesch U.E.A., Leo-Kottler B., Mayer S., Jurklies B., Kellner U., Apfelstedt-Sylla E., Zrenner E., Alexander C., Wissinger B. (2001). OPA1 mutations in patients with autosomal dominant optic atrophy and evidence for semi-dominant inheritance. Hum. Mol. Genet..

[107] Spinazzi M., Cazzola S., Bortolozzi M., Baracca A., Loro E., Casarin A., Solaini G., Sgarbi G., Casalena G., Cenacchi G. (2008). A novel deletion in the GTPase domain of OPA1 causes defects in mitochondrial morphology and distribution, but not in function. Hum. Mol. Genet..

[108] Zanna C., Ghelli A., Porcelli A.M., Karbowski M., Youle R.J., Schimpf S., Wissinger B., Pinti M., Cossarizza A., Vidoni S. (2007). OPA1 mutations associated with dominant optic atrophy impair oxidative phosphorylation and mitochondrial fusion. Brain.

[109] Carelli V., Musumeci O., Caporali L., Zanna C., La Morgia C., Del Dotto V., Porcelli A.M., Rugolo M., Valentino M.L., Iommarini L. (2015). Syndromic parkinsonism and dementia associated with OPA 1 missense mutations. Ann. Neurol..

[110] Lynch D.S., Loh S.H., Harley J., Noyce A.J., Martins L.M., Wood N.W., Houlden H., Plun-Favreau H. (2017). Nonsyndromic Parkinson disease in a family with autosomal dominant optic atrophy due to OPA1 mutations. Neurol. Genet..

[111] Rehman J., Zhang H.J., Toth P.T., Zhang Y., Marsboom G., Hong Z., Salgia R., Husain A.N., Wietholt C., Archer S.L. (2012). Inhibition of mitochondrial fission prevents cell cycle progression in lung cancer. FASEB J..

[112] Xie Q., Wu Q., Horbinski C.M., Flavahan W.A., Yang K., Zhou W., Dombrowski S.M., Huang Z., Fang X., Shi Y. (2015). Mitochondrial control by DRP1 in brain tumor initiating cells. Nat. Neurosci..

[113] Tanwar D.K., Parker D.J., Gupta P., Spurlock B., Alvarez R.D., Basu M.K., Mitra K. (2016). Crosstalk between the mitochondrial fission protein, Drp1, and the cell cycle is identified across various cancer types and can impact survival of epithelial ovarian cancer patients. Oncotarget.

[114] DeTure M.A., Dickson D.W. (2019). The neuropathological diagnosis of Alzheimer’s disease. Mol. Neurodegener..

[115] Wang X., Su B.O., Lee H.G., Li X., Perry G., Smith M.A., Zhu X. (2009). Impaired balance of mitochondrial fission and fusion in Alzheimer’s disease. J. Neurosci..

[116] Reddy P.H., Manczak M., Yin X. (2017). Mitochondria-Division Inhibitor 1 Protects Against Amyloid-beta induced Mitochondrial Fragmentation and Synaptic Damage in Alzheimer’s Disease. J. Alzheimers Dis..

[117] Baek S.H., Park S.J., Jeong J.I., Kim S.H., Han J., Kyung J.W., Jo D.G. (2017). Inhibition of Drp1 Ameliorates Synaptic Depression, Abeta Deposition, and Cognitive Impairment in an Alzheimer’s Disease Model. J. Neurosci..

[118] Joshi A.U., Saw N.L., Shamloo M., Mochly-Rosen D. (2017). Drp1/Fis1 interaction mediates mitochondrial dysfunction, bioenergetic failure and cognitive decline in Alzheimer’s disease. Oncotarget.

[119] Xie H., Guan J., Borrelli L.A., Xu J., Serrano-Pozo A., Bacskai B.J. (2013). Mitochondrial alterations near amyloid plaques in an Alzheimer’s disease mouse model. J. Neurosci..

[120] Poewe W., Seppi K., Tanner C.M., Halliday G.M., Brundin P., Volkmann J., Lang A.E. (2017). Parkinson disease. Nat. Rev. Dis. Primers.

[121] Deng H., Wang P., Jankovic J. (2018). The genetics of Parkinson disease. Ageing Res. Rev..

[122] Lutz A.K., Exner N., Fett M.E., Schlehe J.S., Kloos K., Lämmermann K., Brunner B., Kurz-Drexler A., Vogel F., Reichert A.S. (2009). Loss of Parkin or PINK1 Function Increases Drp1-dependent Mitochondrial Fragmentation. J. Biol. Chem..

[123] Dagda R.K., Cherra S.J., Kulich S.M., Tandon A., Park D., Chu C.T. (2009). Loss of PINK1 Function Promotes Mitophagy through Effects on Oxidative Stress and Mitochondrial Fission. J. Biol. Chem..

[124] Cui M., Tang X., Christian W.V., Yoon Y., Tieu K. (2010). Perturbations in Mitochondrial Dynamics Induced by Human Mutant PINK1 Can Be Rescued by the Mitochondrial Division Inhibitor mdivi-1*. J. Biol. Chem..

[125] Wang X., Su B., Liu W., He X., Gao Y., Castellani R.J., Perry G., Smith M.A., Zhu X. (2011). DLP1-dependent mitochondrial fragmentation mediates 1-methyl-4-phenylpyridinium toxicity in neurons: Implications for Parkinson’s disease. Aging Cell.

[126] Rappold P.M., Cui M., Grima J.C., Fan R.Z., De Mesy-Bentley K.L., Chen L., Zhuang X., Bowers W.J., Tieu K. (2014). Drp1 inhibition attenuates neurotoxicity and dopamine release deficits in vivo. Nat. Commun..

[127] Zhang Z., Liu L., Jiang X., Zhai S., Xing D. (2016). The Essential Role of Drp1 and Its Regulation by S-Nitrosylation of Parkin in Dopaminergic Neurodegeneration: Implications for Parkinson’s Disease. Antioxidants Redox Signal..

[128] Filichia E., Hoffer B., Qi X., Luo Y. (2016). Inhibition of Drp1 mitochondrial translocation provides neural protection in dopaminergic system in a Parkinson’s disease model induced by MPTP. Sci. Rep..

[129] Zhu D., Kennerson M.L., Walizada G., Zuchner S., Vance J.M., Nicholson G.A. (2005). Charcot-Marie-Tooth with pyramidal signs is genetically heterogeneous: Families with and without MFN2 mutations. Neurology.

[130] Larrea D., Pera M., Gonnelli A., Quintana–Cabrera R., Akman H.O., Guardia-Laguarta C., Velasco K.R., Area-Gomez E., Bello F.D., De Stefani D. (2019). MFN2 mutations in Charcot–Marie–Tooth disease alter mitochondria-associated ER membrane function but do not impair bioenergetics. Hum. Mol. Genet..

[131] Spiegel R., Saada A., Flannery P.J., Burté F., Soiferman D., Khayat M., Eisner V., Vladovski E., Taylor R.W., Bindoff L.A. (2016). Fatal infantile mitochondrial encephalomyopathy, hypertrophic cardiomyopathy and optic atrophy associated with a homozygousOPA1mutation. J. Med Genet..

[132] Gerber S., Charif M., Chevrollier A., Chaumette T., Angebault C., Kane M.S., Paris A., Alban J., Quiles M., Delettre C. (2017). Mutations in DNM1L, as in OPA1, result in dominant optic atrophy despite opposite effects on mitochondrial fusion and fission. Brain.

[133] Koch J., Feichtinger R.G., Freisinger P., Pies M., Schrödl F., Iuso A., Sperl W., Mayr J.A., Prokisch H., Haack T.B. (2016). Disturbed mitochondrial and peroxisomal dynamics due to loss of MFF causes Leigh-like encephalopathy, optic atrophy and peripheral neuropathy. J. Med. Genet..

[134] Panda I., Ahmad I., Sagar S., Zahra S., Shamim U., Sharma S., Faruq M. (2020). Encephalopathy due to defective mitochondrial and peroxisomal fission 2 caused by a novel MFF gene mutation in a young child. Clin. Genet..

[135] Yu T., Fox R.J., Burwell L.S., Yoon Y. (2005). Regulation of mitochondrial fission and apoptosis by the mitochondrial outer membrane protein hFis1. J. Cell Sci..

[136] Bartsakoulia M., Pyle A., Troncoso-Chandía D., Vial-Brizzi J., Paz-Fiblas M.V., Duff J., Griffin H., Boczonadi V., Lochmüller H., Kleinle S. (2018). A novel mechanism causing imbalance of mitochondrial fusion and fission in human myopathies. Hum. Mol. Genet..

[137] Charif M., Wong Y.C., Kim S., Guichet A., Vignal C., Zanlonghi X., Bensaid P., Procaccio V., Bonneau D., Amati-Bonneau P. (2021). Dominant mutations in MIEF1 affect mitochondrial dynamics and cause a singular late onset optic neuropathy. Mol. Neurodegener..

[138] Bitoun M., Maugenre S., Jeannet P.-Y., Lacène E., Ferrer X., Laforêt P., Martin J.-J., Laporte J., Lochmüller H., Beggs A.H. (2005). Mutations in dynamin 2 cause dominant centronuclear myopathy. Nat. Genet..

[139] Züchner S., Noureddine M., Kennerson M., Verhoeven K., Claeys K., De Jonghe P., Merory J., Oliveira S.A., Speer M.C., Stenger J.E. (2005). Mutations in the pleckstrin homology domain of dynamin 2 cause dominant intermediate Charcot-Marie-Tooth disease. Nat. Genet..

[140] Fabrizi G.M., Ferrarini M., Cavallaro T., Cabrini I., Cerini R., Bertolasi L., Rizzuto N. (2007). Two novel mutations in dynamin-2 cause axonal Charcot-Marie-Tooth disease. Neurology.

